# The Differential Effects of Anger on Trust: A Cross-Cultural Comparison of the Effects of Gender and Social Distance

**DOI:** 10.3389/fpsyg.2020.597436

**Published:** 2020-12-23

**Authors:** Keshun Zhang, Thomas Goetz, Fadong Chen, Anna Sverdlik

**Affiliations:** ^1^Department of Psychology, Qingdao Psychological and Mental Health Research Institute, Qingdao University, Qingdao, China; ^2^Graduate School of Decision Sciences, University of Konstanz, Konstanz, Germany; ^3^Department of Developmental and Educational Psychology, Faculty of Psychology, University of Vienna, Vienna, Austria; ^4^Neuromanagement Lab, School of Management, Zhejiang University, Hangzhou, China; ^5^Department of Educational and Counselling Psychology, McGill University, Montreal, QC, Canada

**Keywords:** trust, anger, gender, social distance, cross-cultural

## Abstract

Accumulating empirical evidence suggests that anger elicited in one situation can influence trust behaviors in another situation. However, the conditions under which anger influences trust are still unclear. The present study addresses this research gap and examines the ways in which anger influences trust. We hypothesized that the social distance to the trustee, and the trusting person’s gender would moderate the effect of anger on trust. To test this hypothesis, a study using a 2 (Anger vs. Control) × 2 (Low vs. High social distance) × 2 (Men vs. Women) factorial design was conducted in Germany (*N* = 215) and in China (*N* = 310). Results reveal that in both countries men’s trust behavior was not influenced by the manipulations (i.e., anger and social distance). The pattern for women, however, differed by country. In Germany, women’s trust to a stranger (i.e., high social distance) was increased by anger; while in China, women’s trust to someone who they have communicated with (i.e., low social distance) was increased by anger. These results indicate that women’s trust levels seem to be more context-sensitive than men’s.

## Introduction

Trust is a fundamental component of human relationships, often playing a role in perpetuating cooperative relationships among groups and individuals (e.g., Fukuyama, 1995; Hoffman et al., 1998; Fehr, 2009; Algan and Cahuc, 2013). Trust is defined as “a psychological state comprising the intention to accept vulnerability based upon positive expectations of the intentions or the behavior of another” (Rousseau et al., 1998, p. 395), and has been viewed as a rational act based on thorough cognitive assessments (Dunning et al., 2012; Schlösser et al., 2015). However, recent accumulating empirical evidence suggests that trust behaviors might be influenced by emotions (e.g., Dunning et al., 2012; Engelmann and Fehr, 2013). The specific ways in which emotions influence trust, however, are still unclear. For this reason, we will examine the way in which anger influences trust in the present work. In doing so, we will explore the role of gender and social distance as factors shaping the effects of anger on trust. Specifically, as increasing evidence indicates that women more frequently use social cues to form their trust than men (e.g., Croson and Gneezy, 2009; Rand et al., 2016), we will investigate the moderating role of gender on the relationship between anger and trust. In addition to gender, we will take social distance into account as previous research points to the importance of perceived social distance from the trustee on people’s trust behavior (Dunn and Schweitzer, 2005; Forgas, 1995). Thus, in the present work we will investigate the effects of anger on trust, depending on the trusting person’s gender (Croson and Buchan, 1999; Buchan et al., 2008) and perceived social distance from the trustee. Furthermore, to address the issue of the generalizability of the assumed functional associations, we test our predictions with two cross-cultural samples from Germany and China, which represent substantially different cultural backgrounds.

## The Influence of Anger on Trust

For a long time, trust has been depicted as a deliberated act based on thorough cognitive calculations, by assessing the desirability and likelihood of its consequence (e.g., Williamson, 1993; Fetchenhauer and Dunning, 2009). However, this does not imply that decision makers are devoid of emotions or immune to their influence in their trust taking process (Dunning et al., 2012; Engelmann and Fehr, 2013). In the following sections we will briefly review theoretical frameworks and empirical evidence outlining how and when anger influences trust.

### The Relationship Between Anger and Trust

In the present work, we use the *Appraisal Tendency Framework* (ATF; Lerner and Keltner, 2000, 2001; Han et al., 2007) as a theoretical model to understand how incidental anger influences trust. The ATF assumes that “each emotion activates a cognitive predisposition to appraise future events in line with the central-appraisal dimensions that triggered the emotion” (Lerner and Keltner, 2000, p. 477). The ATF allows a precise prediction of the differential impact of discrete emotions on particular judgments due to their link to emotion-specific appraisal tendencies. To yield strong influences, the emotion’s central appraisal content must be thematically linked to the decision-making topic (Lerner and Keltner, 2001). Previous studies have identified three central dimensions of emotions that could be used to distinguish the effect of anger on judgments and choices. These dimensions are *control, certainty* appraisal, and *associated motivation* (e.g., Smith and Ellsworth, 1985; Lerner and Keltner, 2001; Dunn and Schweitzer, 2005; Carver and Harmon-Jones, 2009). More specifically, *control* is the degree to which events seem to be brought by individual agency (high) vs. situational agency (low), *certainty* is the degree to which future events seem predictable (high) vs. unpredictable (low), *associated motivation* is the urge to approach or avoid a stimulus (Lerner and Keltner, 2000; Angus et al., 2015). Anger is characterized by high appraisals of both certainty and control, which promote people to perceive new situations as being certain and highly controllable and therefore also involving less risk (Lerner and Keltner, 2000). Furthermore, anger is also associated with approach motivation (Carver and Harmon-Jones, 2009) which facilitates the tendency to pursue rewarding stimuli, even in the face of certain risks associated with these stimuli (Angus et al., 2015). In the context of trust-related decisions, the above three appraisal dimensions of anger are thought to be particularly influential because of their close association with cognitive evaluations for determining trust decisions. Hence, we predict that angry people will demonstrate less risk aversion and more reward seeking, and thus trust others more.

### The Moderating Role of the Social Distance From the Trustee

Under what circumstances can anger influence trust? It seems that there is no simple answer. There are two different cognitive processes, namely heuristic processes and analytic processes, which are proposed by the heuristic-analytic theory (Evans, 2003, 2006) and the affect infusion model (AIM, Forgas, 1995). Heuristic processes are unconscious, rapid, automatic, and high capacity, and generate selective representations of problem content. Analytic processes, on the other hand, are conscious, slow and deliberative, and inferences or judgments are derived from these representations (Evans, 2006, 2008). These two types of cognitive processes might determine whether affect influences judgment and decision making. According to these models, affect influences judgments when people use heuristic processes, but not analytic processes (Forgas, 1995; Evans, 2008). When people involve in heuristic processing they tend to make judgments consistent with the ATF, making their decisions based on affective information (Lerner and Keltner, 2001; Slovic et al., 2007). For example, Schwarz and Clore (1988) have argued that when people make decisions, they unconsciously ask themselves “how do I feel about the decision?”. To answer this question, one may use the appraisal dimensions of one’s unrelated emotions to inform the decision at hand. However, when people use analytic processing, affect does not influence the decision (Forgas, 1995). The analytic processing is often used when there is strong and specific motivation to achieve a particular judgmental outcome, in which one’s preferences, but not emotions, may guide their inferences (Forgas, 1995; Evans, 2006, 2008). In the present study, we investigate when affect heuristic takes place in the trust process.

One factor that is suggested to moderate the relationship between emotions and trust is the social distance from the trustee (Forgas, 1995; Dunn and Schweitzer, 2005). Social distance is a measure of the closeness between players in a strategic interaction (Akerlof, 1997). The decision makers have no available information about the trustee who is an absolute stranger, and consequently they have no specific motivational objectives and limited information for their decisions. Thus, decisions may be based on irrelevant associations with their current emotions (Clore et al., 1994; Schwarz and Clore, 2003), as the available information at hand. The trusting person under these circumstances may follow the heuristic information processing to form their trust, which will consequently be largely influenced by the affect heuristic (Schwarz and Clore, 2003; Dunn and Schweitzer, 2005).

However, when the trusting person has some information about the trustee, for example, they know personal information about trustee, the trusting person might be more motivated to make an informed decision about this person. Thus, the decision maker may engage in the analytic information processing to analyze their accumulated information from past communications, in addition to the information from the anger heuristic, and then make their trust decisions based on their analysis (Buchan et al., 2006; Slovic et al., 2007). It is a relatively slower and more controlled process, as compared with the affective heuristic processing. As a result, emotions are likely to exert little influence on trust. In the present study, we examine the influence of anger on trust behavior toward strangers (i.e., no communication at all between the two parties, i.e., high social distance), and toward someone who the participant has communicated with (i.e., low social distance). We expect the social distance of the trustee to moderate the effects of anger on trust, and following the above explanation we assume that anger influences trust toward strangers more than trust toward someone who the person has communicated with.

### The Moderating Role of Gender

In additional to the social distance of the trustee, gender is another important factor that is suggested to moderate the effects of anger on trust (Croson and Gneezy, 2009). According to the social role theory, men are more focused on the task whereas women tend to be more socially oriented (Anderson and Blanchard, 1982; Eagly and Wood, 1991). In line with this theory, a large body of empirical evidence in the field of trust suggests that women’s trust varies to a greater extent than men’s based on heuristics perceived during interactions (see review, Croson and Gneezy, 2009; Rand et al., 2016), such as the gender information (Buchan et al., 2008) or descriptive information (Eckel and Wilson, 2003) of the trustee.

Furthermore, increasing evidence indicates that emotion is one of the essential heuristics which human apply to determine one’s trust (see review, Dunning et al., 2012; Engelmann and Fehr, 2013). In the present study, we aim to examine whether a gender difference exists in applying affect heuristic in the formation of trust behavior. As women are more sensitive than men to social heuristics in forming trust behavior, we assume that gender plays a moderating role in the effect of anger on trust. More specifically, we expect women, but not men, to apply their anger as a heuristic in determining their trust-related actions. Therefore, we assume that women’s trust is influenced by anger more than men’s.

### Cultural Influences

When considering the underlying mechanisms of how anger influences trust, cultural influences seem to be a pertinent issue, especially when comparing individualistic and collectivistic cultures (e.g., Hofstede, 2001; Beisswingert et al., 2015). However, only little cross-cultural work concerning the effects of anger on trust has been published.

Even though there is no question that there are cultural differences in the frequency and intensity of emotions (van Hemert et al., 2007; Matsumoto et al., 2008), the general functional mechanisms of how emotions influence human behavior are supposed to be the same in the individualistic and collectivistic cultures (Pekrun, 2006; Lerner et al., 2015). For example, research in economics has investigated the influence of incidental emotion on the macro level of behavior. Based on the hypothesis that people are happier on sunny days, economists found a positive correlation between the amount of sunshine on a given day and stock market performance across 26 countries, which including both individualistic (e.g., United States and Germany) and collectivistic (e.g., Japan and Thailand) countries (Hirshleifer and Shumway, 2003; Kamstra et al., 2003). Correspondingly, research in psychology has begun to study incidents of emotion carryover at the micro level of behavior (using the individual as the unit of analysis); participants from both Germany and China are more risk-taking when they are angry, although German participants perceives higher level of anger than Chinese in doing the same task (Beisswingert et al., 2015). Based on the cross-cultural evidences about micro-level and macro-level behavior of humans, these studies make a promising connection between emotions and human behavior.

Instead of focusing on absolute mean levels of anger and social distance, this study will investigate the effects of anger on trust behavior and the proposed moderating roles of gender and social distance in this relationship. Furthermore, we will explore whether these relational and functional associations are cross-culturally valid. For this purpose, we use samples from Germany and China, which represent differing cultures with respect to mean levels in the variables of interest. We expect that the moderating effects of gender and social distance on the relationship between anger and trust to be cross-culturally generalizable.

### The Present Research

In the present studies, we will investigate the influence of anger on trust. Based on the gender differences in applying heuristic processing in social interactions and the AIM, we predict that the trusting person’s gender and the social distance of the trustee moderate the effects of anger on trust (*Hypothesis 1*). Moreover, we will explore the cross-cultural generalizability (Germany vs. China) of the proposed effects of anger, gender and social distance on trust. We hypothesize that the moderating effects of the trusting person’s gender and the social distance of the trustee on the relationship between anger and trust is cross-culturally generalizable (*Hypothesis 2*).

## Study 1

Study 1 aimed at investigating the effect of anger on trust behavior within a German sample, by applying the adapted and tested “Autobiographical Emotional Memory Task” (AEMT, Strack et al., 1985; Mills and D’Mello, 2014) in the pilot study to arouse anger (see Appendix 1). Furthermore, we explored the role of gender and the social distance of the trustee in the effect of anger on trust (*Hypothesis 1*).

### Method

#### Participants and Data Collection

A total of 216 German university students voluntarily participated in this study. One participant who was not native German and could not follow the German instructions in the experiment was excluded from further data analyses. The final sample consisted of 215 participants (46.5% female), with an average age of *M* = 20.97 years (*SD* = 2.50, range: 18–40). The participants were recruited using the online recruiting system ORSEE (Greiner, 2015). Their participation was compensated by a fixed show-up fee (3 €), plus payment according to their individual decisions in the trust game (theoretical range: 0–12 €), which on average resulted in a pay of 7.70 €.

#### Experimental Design and Procedures

##### Experimental design

This study used a 2 (Anger vs. Control) × 2 (Low vs. High social distance) × 2 (Male vs. Female) factorial design. Participants were randomly assigned to one of the four experimental conditions, with balanced gender. First, social distance of the trustee was manipulated and then anger. Then, participants were paired to play the trust game. The response time in the trust game was recorded. Anger, perceived social distance of trustee, as well as socio-demographic variables (e.g., general trust belief in other people, gender, age, program of study, monthly disposable income, previous experience with computer-games) were measured after playing the trust game. The experiment was programmed using z-Tree (Fischbacher, 2007).

##### Chatting task

The social distance of the trustee was manipulated via an online chatting task. In the chat condition, four anonymous participants in each session were in one chatting group. They could chat about either one of the three suggested topics (your favorite sports, your favorite holiday or a memorable birthday celebration; Buchan et al., 2006; Fiedler et al., 2011) or any topics they preferred except their names. They had 5 min to talk with their group members via the chatting program on the computer. In the no chat condition, participants had no communication with one another prior to the anger manipulation. Notice that the communications in the chat condition could not have been strategy-relevant to the trust game, as participants did not know they were going to play a trust game later (Buchan et al., 2006). In the chat condition, participants were then informed that they will play the trust game with someone random from their chatting group, while in the no-chat condition, the game partner was someone random from the same session of experiment.

##### Autobiographical emotional memory task (AEMT)

This study adopted the Autobiographical Emotional Memory Task (AEMT; Mills and D’Mello, 2014; Strack et al., 1985) to elicit anger. We further specified the sources of anger as another person. Participants in the anger condition were asked to describe an angry event with the following instruction: “*Please describe in detail the one situation caused by another person (not yourself) that has made you the most angry you have ever been in your life, and vividly describe how the event occurred. Please describe it such that a person reading the description would become [angry] just from hearing about the situation.”* While in the control condition, participants were asked to *“Describe in detail the mundane events of the previous day”* (Bodenhausen et al., 2000). Participants typed their responses on the computer and the content of their responses was stored for offline analysis. Participants were suggested to finish writing in 6 min, and they could continue to write for an extra 2 min, if necessary. We conducted a pilot study as a manipulation check to ensure that the anger was successfully aroused in the experimental condition as compared to a control condition (see Appendix 1).

##### The trust game

An investment game (Berg et al., 1995) was applied to assess participants’ trust. In this game, there are two players (*A* and *B*); both are anonymous and randomly paired to each other. They are informed that they will interact with each other only once. Both *A* and *B* will receive an initial endowment of 30 points (1 point = 0.10 €) from the experimenter. *A* then has the opportunity to give a portion of their points to *B*. *A* can choose whether to send 0, 10, 20, or 30 points to *B*. Whatever amount *A* decides to send to *B* will be tripled by the experimenter before it is passed on to *B*. *B* then has the option of returning any amount between zero and their total amount to *A*. For example, if *A* sends 10 points, they are tripled to 30 points before they are passed on to *B*. Then *B* possesses 60 points (30 points own endowment + 30 tripled points) and can choose any back transfer from 0 to 60 points. All participants start the game as player *A*. Only after they finish making the decision of *A*, they are instructed to play the role of *B* as well (Burks et al., 2003).

The final payoff of player *A* corresponds to the initial endowment minus the transfer to *B*, plus the back transfer from *B*. The final payoff of player *B* is given by his initial endowment plus the tripled transfer of *A*, minus the back transfer to *A*. At the end of the experiment, we randomly choose one of participants in each session to roll a die to decide which role (as player *A* or *B*) of them would be paid in this game. The earned points are exchanged into real money according to a publicly announced exchange rate.

#### Variables and Study Measures

##### Anger

Applying the subscales of the Differential Emotion Scale (Izard et al., 1974; German version: Merten and Krause, 1993), anger was assessed after the trust game as a manipulation check. The subscale consists of three items (enraged, angry, and mad). Participants’ anger was assessed by their ratings on a five-point intensity rating scale ranging from 0 *not at all* to 4 *very strong*. The internal consistency of the anger scale was *a* = 0.94.

##### Social distance

The social distance that participants perceived toward their game partner in the trust game was assessed with the Inclusion of Other in the Self (IOS) Scale (Aron et al., 1992). It was measured as a manipulation check. Participants rated on a set of seven Venn-like diagrams, ranging from 1 (small social distance) to 7 (large social distance)^1^. The social distance that participants perceived as player *A* was measured.

### Results

#### Anger

**FIGURE 1:**
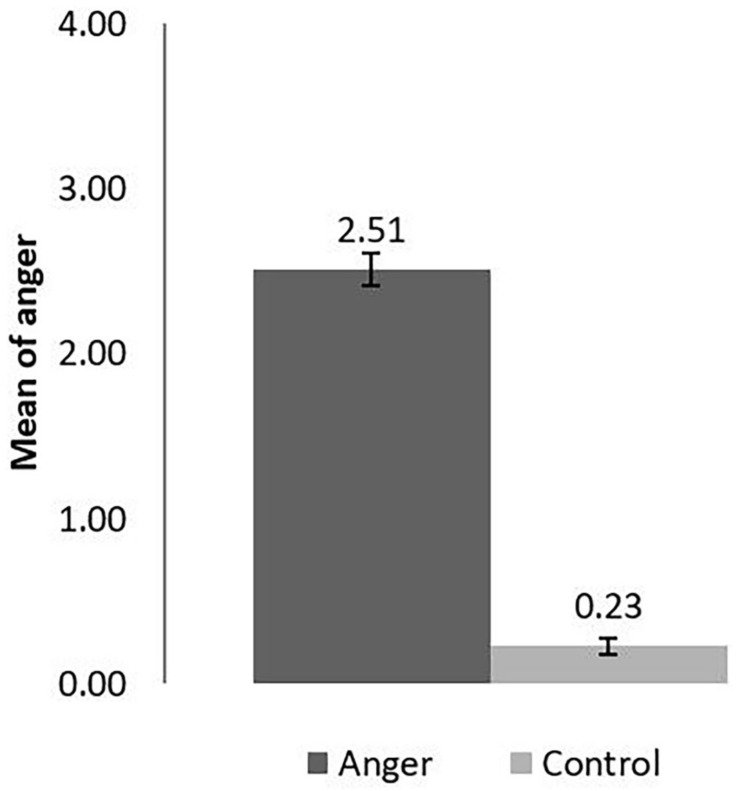
Anger rating of the AEMT in the angry and control condition in Study 1. The error bar is the Standard error.

Following the experimental manipulation, participants in the anger condition (*M* = 2.51, *SD* = 1.09) showed significantly higher levels of anger than the participants in the control condition [*M* = 0.23, *SD* = 0.51, *t*(213) = 19.36, *p* < 0.001, *d* = −2.64; see Figure 1]. Therefore, results from anger assessment showed the experimental manipulation was successful.

#### Trust

The descriptive result of trust is shown in Table 1. We used regression to assess the effects of emotion (anger/control), social distance (low/high social distance) and gender on trust [anger = 1, control = 0; low social distance (LSD) = 1, high social distance (HSD) = 0; male = 1, female = 0], and controlled the general trust belief in other people as covariate. This regression was significant, *R*^2^ = 0.065, *F*(8,206) = 1.80, *p* = 0.048, $\eta_{\text{p}}^{2}$ = 0.07, 1−β = 0.76. The regression coefficients for emotion (β = 6.11, *t* = 2.73, *p* = 0.026, 95% CI [0.73, 11.49]) and gender (β = 6.82, *t* = 2.53, *p* = 0.012, 95% CI [1.51, 12.13]) were significant, while the coefficient for social distance (β = 3.84, *t* = 1.38, *p* = 0.168, 95% CI [−1.63, 9.31]) was not significant. The two-way interaction between emotion and social distance (β = −8.25, *t* = −2.19, *p* = 0.030, 95% CI [−15.68, −0.81]) was significant, as well as the interaction between emotion and gender was marginal significant (β = −6.88.07, *t* = −1.88, *p* = 0.062, 95% CI [−14.10, 0.35]), qualified by a significant three-way interaction between emotion, social distance and gender (β = 11.89, *t* = 2.30, *p* = 0.023, 95% CI [1.69, 22.09]). The regression coefficient for the general trust belief in other people was not significant (β = 0.50, *t* = 0.83, *p* = 0.406, 95% CI [−0.68, 1.68]).

**TABLE 1:** Means and standard deviations of trust by emotion, social distance and gender in Study 1.

			*M*	*SD*	*N*
Female	HSD	Control	12.86	7.17	21
		Anger	18.89	8.47	27
	LSD	Control	16.80	9.45	25
		Anger	14.44	7.51	27
Male	HSD	Control	20.00	10.17	30
		Anger	18.97	9.00	29
	LSD	Control	17.04	9.93	27
		Anger	20.00	11.65	29

**Participants could send either 0, 10, 20, or 30 points (1 point* = *0.10 €) to their game partner in the trust game. Low social distance: LSD, high social distance: HSD.**

**FIGURE 2:**
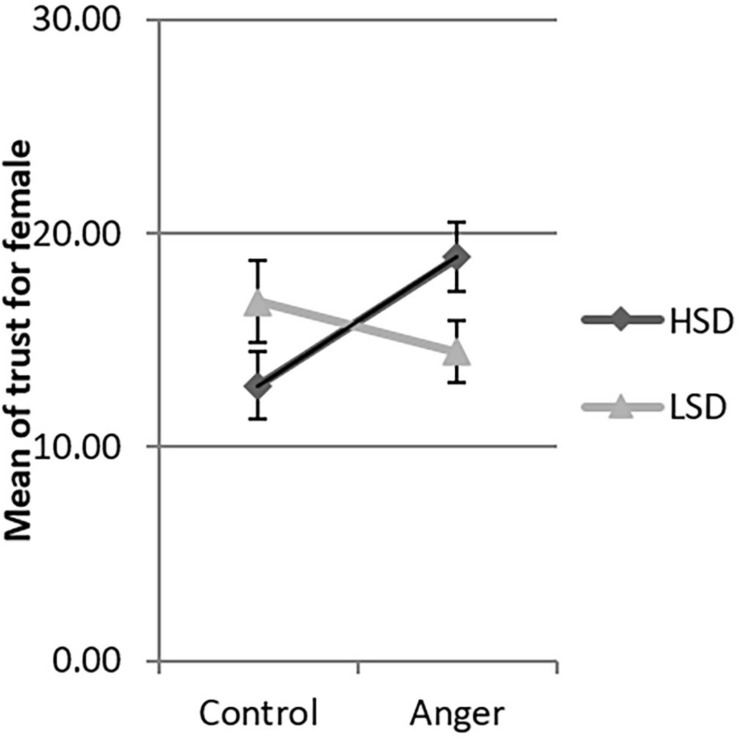
Mean of trust of female the four experimental conditions in Study 1. LSD, low social distance; HSD, high social distance. Error bar is the standard error.

**FIGURE 3:**
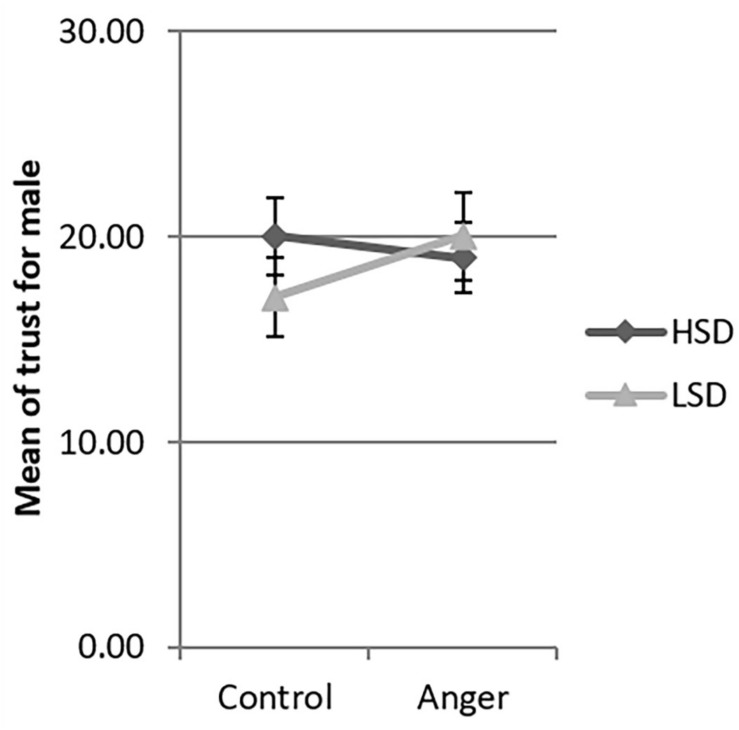
Mean of trust of male across the four experimental conditions in Study 1. LSD, low social distance; HSD, high social distance. Error bar is the standard error.

Since the three-way interaction between emotion, social distance and gender was significant, we tested the interaction between emotion and social distance by gender. There was significant interaction between emotion and social distance for females [β = −8.25, *F*(1,206) = 4.79, *p* = 0.030], but not for males [β = 3.65, *F*(1,206) = 1.07, *p* = 0.302]. We used the simple slopes method (Aiken et al., 1991) to investigate this interaction in detail. Women in the HSD condition demonstrated more trust in the anger condition than in the control condition (β = 6.11, *t* = 2.73, *p* = 0.026, 95% CI [0.73, 11.49], see Figure 2). However, for women in the LSD condition, the levels of trust were not significantly different between the control and anger conditions (β = −2.14, *t* = −0.82, *p* = 0.414, 95% CI [−7.23, 3.02]). In both the HSD and LSD conditions, the trust of men was not significantly different across the anger and the control conditions (β = −0.77, *t* = −0.31, *p* = 0.756, 95% CI [−5.62, 4.07]; β = 2.88, *t* = 1.15, *p* = 0.252, 95% CI [−2.06, 7.82] see Figure 3).

### Discussion

The results of this study support the predicted three-way interaction between anger, gender, and social distance on trust. In line with research on the affect heuristic, women in anger condition increased their trust when the trustee was an absolute stranger, but this effect faded away when women had prior chatting experience with the trustee. On one hand, we argue that reducing social distance might have lead women to use analytical processing to form their trust. On the other hand, if the anger experience was caused by a close person, the spillover effect of a betrayal feeling might also decrease women’s trust to people with low social distance (Beisswingert et al., 2016). Men’s trust was influenced neither by anger nor by the social distance of the trustee. Consistent with Croson and Gneezy (2009), these results supported that women’s trust behavior is more context-sensitive than men’s. Women, but not men, might form their trust based on their current experienced emotion, as well the perceived social distance of the trustee. In conclusion, the results of Study 1 were in line with our expectations and provided supporting evidence for *Hypotheses 1*. In Study 2, we aim to test the cross-cultural generalizability of our conclusions from study 1, applying the same procedures to a sample from a collectivist culture.

## Study 2

The objective of Study 2 was to test *Hypothesis 2* which proposed that the moderating effects of gender and the perceived social distance of the trustee on the relationship between anger and trust was cross-culturally generalizable. To achieve this goal, we aimed at replicating Study 1 with a Chinese sample to allow for cross-cultural comparisons.

### Method

#### Participants and Data Collection

A total of 310 (57.1% female) Chinese university students voluntarily participated in the study. The average age was *M* = 20.76 years (*SD* = 1.83, range: 18–28). The participants were randomly assigned to each experimental condition. Their participation was compensated by a fixed show-up fee (12 RMB) plus payment according to their individual decisions in the trust game, which on average resulted in a pay of 31.69 RMB for a 30-min experiment^2^.

#### Experimental Design, Procedures, and Variables

In Study 2 we applied the same 2 (Anger vs. Control) × 2 (Low vs. High social distance) × 2 (Male vs. Female) factorial design, procedures and measures in the Chinese samples as we did in Study 1 in the German sample. Thus, following the manipulations of social distance and emotion, the trust game was used once again to measure the trust behavior. The response time in the trust game was also recorded. Anger, perceived social distance of trustee, as well as social-demographic variables were assessed after playing trust game. A Chinese version of the anger subscale of the Differential Emotions Scale was applied from Beisswingert et al. (2015). The internal consistency of the anger subscale was α = 0.91 at pre-rating and α = 0.82 at post-rating. All the other measures (chatting task, AEMT, investment game, general trust belief in other people, as well as social-demographic variables) were subjected to a multiple stage translation process, including independent translations by two professional translators, as well as comparisons, revisions, and a pilot test with Chinese students (see Appendix 2). In accordance with the Chinese currency, one experimental point in this study was 0.4 RMB and the theoretical profit from the trust game was 0–48 RMB.

### Results

#### Anger

**FIGURE 4:**
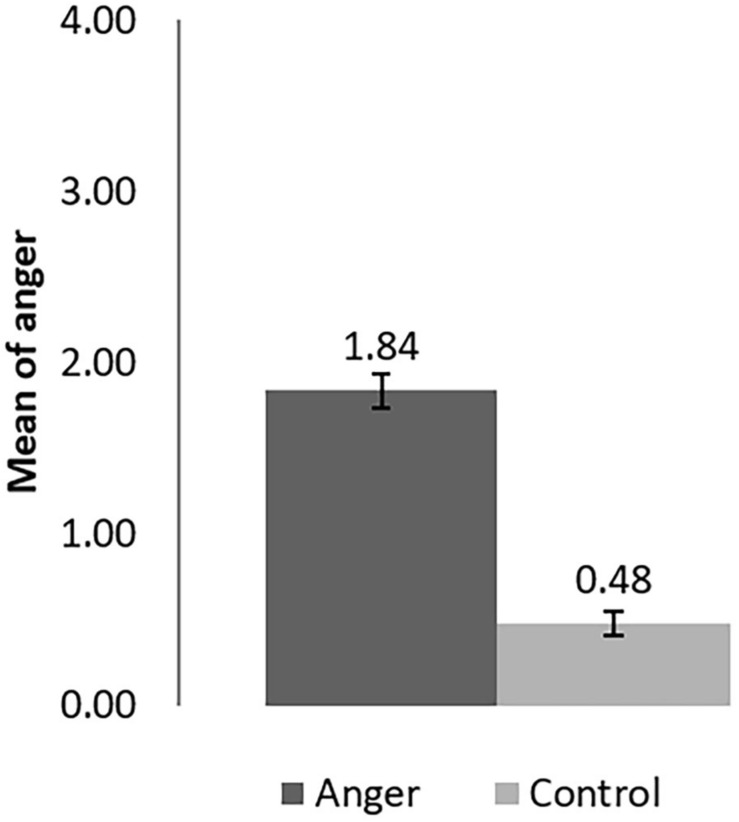
Anger rating of the AEMT in the angry and control condition in Study 2. The error bar is the Standard error.

Participants in the anger condition (*M* = 1.87, *SD* = 1.21) showed significantly higher levels of anger than the participants in the control condition [*M* = 0.48, *SD* = 0.80, *t*(308) = 11.82, *p* < 0.001, *d* = −1.34; see Figure 4]. Therefore, results from anger assessment showed that the experimental manipulation was successful.

#### Trust

The descriptive results of trust are shown in Table 2. We used regression to assess the effects of emotion (anger/control), social distance (low/high social distance) and gender on trust [anger = 1, control = 0; low social distance (LSD) = 1, high social distance (HSD) = 0; male = 1, female = 0], and controlled the general trust belief in other people as covariate. This regression was significant, *R*^2^ = 0.11, *F*(8,301) = 4.55, *p* = 0.000, $\eta_{\text{p}}^{2}$ = 0.11, 1−β = 0.99. The regression coefficient for social distance was significant (β = −4.42, *t* = −2.40, *p* = 0.017, 95% CI [−8.04, −0.79]), while the coefficients for emotion (β = −1.34, *t* = −0.87, *p* = 0.376, 95% CI [−4.31, 1.63]) and gender (β = 1.88, *t* = 1.04, *p* = 0.301, 95% CI [−1.70, 5.48]) were not significant. The two-way interactions between emotion and social distance (β = 5.52, *t* = 2.17, *p* = 0.031, 95% CI [0.52, 10.53]), as well as between social distance and gender were significant (β = 7.50, *t* = 2.76, *p* = 0.006, 95% CI [2.15, 12.86]), qualified by a significant three-way interaction between social distance, emotion and gender (β = −10.43, *t* = −2.74, *p* = 0.007, 95% CI [−17.92, −2.93]). The regression coefficient for the general trust belief in other people was also significant (β = 1.43, *t* = 2.78, *p* = 0.006, 95% CI [0.42, 2.45]).

**TABLE 2:** Means and standard deviations of trust by emotion, social distance and gender in Study 2.

			*M*	*SD*	*N*
Female	HSD	Control	17.74	7.76	53
		Anger	16.61	7.88	62
	LSD	Control	13.33	6.61	30
		Anger	17.81	7.93	32
Male	HSD	Control	19.68	8.36	31
		Anger	20.50	9.32	40
	LSD	Control	22.65	8.28	34
		Anger	19.29	9.00	28

**Participants could send either 0, 10, 20, or 30 points (1 point* = *0.40 RMB) to their game partner in the trust game. Low social distance: LSD, high social distance: HSD.**

**FIGURE 5:**
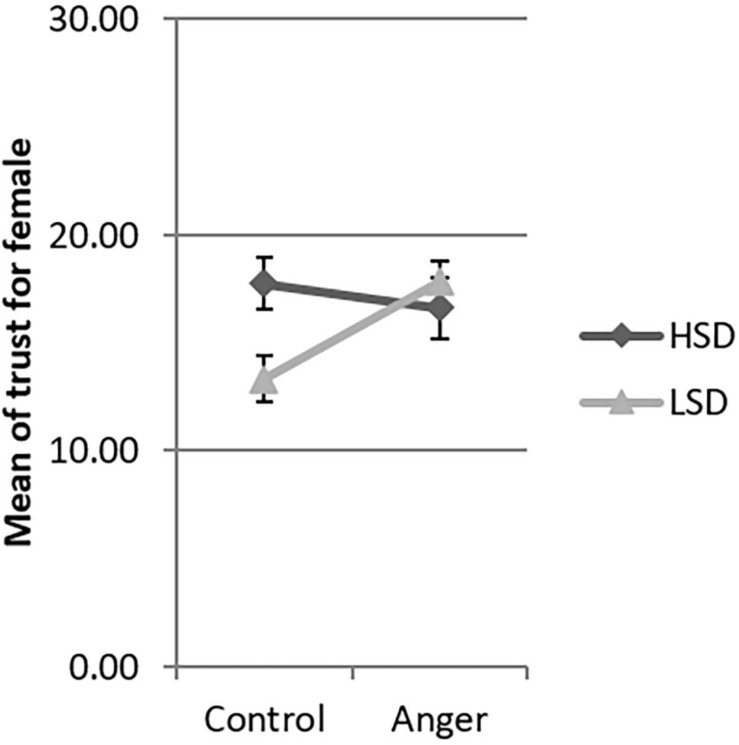
Mean of trust of female across the four experimental conditions in Study 2. LSD, low social distance; HSD, high social distance. Error bar is the standard error.

**FIGURE 6:**
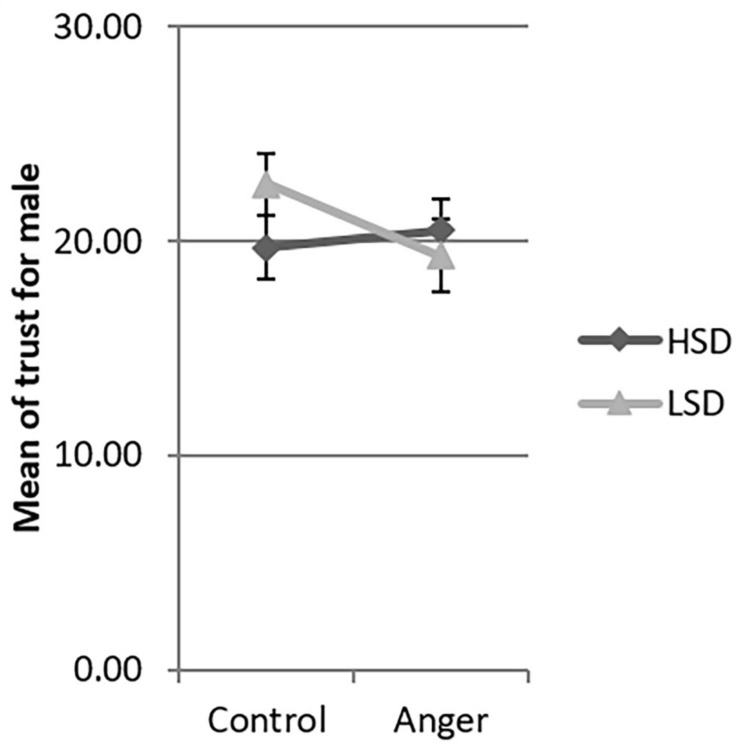
Mean of trust of male across the four experimental conditions in Study 2. LSD, low social distance:; HSD, high social distance. Error bar is the standard error.

Because the three-way interaction between emotion, social distance, and gender was significant, we tested the differential interaction between social distance and emotion by gender. There was a significant interaction between emotion and social distance for females [β = 5.52, *F*(1,301) = 4.71.18, *p* = 0.031], but not for males [β = −4.91, *F*(1,301) = 3.00, *p* = 0.084]. We used the simple slopes method (Aiken et al., 1991) to investigate this interaction in detail. On the one hand, for women in the HSD condition, their trust was not significantly different between the anger and the control conditions (β = −1.34, *t* = −0.89, *p* = 0.376, 95% CI [−4.31, 1.63]). Women in the LSD condition, on the other hand, were more trusting in the anger condition than in the control condition (β = 4.18, *t* = 2.04, *p* = 0.042, 95% CI [0.15, 8.22] see Figure 5). Women in the control condition sent more money in the HSD condition than in the LSD condition (β = −4.42, *t* = −2.40, *p* = 0.017, 95% CI [−8.04, −0.79]), whereas the trust of women in the anger condition was not significantly different between the HSD and LSD conditions (β = 1.10, *t* = 0.63, *p* = 0.530, 95% CI [−2.35, 4.56]). In both the HSD and LSD conditions, the average trust of men was not significantly different between the anger and the control conditions (β = 1.04., *t* = 0.54, *p* = 0.591, 95% CI [−2.76, 4.84]; β = −3.87, *t* = −1.87, *p* = 0.062 95% CI [−7.93, 0.20]; see Figure 6).

### Discussion

In Study 2, the assumed moderating roles of gender and social distance in the effect of anger on trust behavior were supported within a Chinese sample, but in different patterns as compared with in German sample. Consistent with the findings of Study 1, Chinese men’s trust was not influenced by anger nor by the social distance from the trustee. However, the trust pattern of Chinese women was different from the one of German women. Trust was increased by anger when Chinese women had prior chatting experience with the trustee but not when the trustees were absolute strangers, which was not in line with our expectation. Furthermore, Chinese women sent more money to the absolute strangers than to someone who they had chatted with. These results are consistent with previous work in China demonstrating that Chinese individuals trust outgroup members significantly more than ingroup members when the social distance is manipulated through the creation of artificial groups, a circumstance which we used in this experiment as well (Buchan et al., 2006). Overall, results of this study again indicate that women, but not men, probably form their trust based on their present emotional state as well the perceived social distance of the trustee. In conclusion, the results of Study 2 were partially in line with our expectations in *Hypotheses 2*.

## General Discussion

The willingness to trust others is fundamental to economic and social life (Fukuyama, 1995; Hoffman et al., 1998; Fehr, 2009; Algan and Cahuc, 2013). There is accumulating evidence suggesting that trust behaviors involve emotions. This emotional aspect of trust, however, is rarely investigated as compared to the well-researched cognitive aspect (Dunning et al., 2012; Engelmann and Fehr, 2013). This study investigated how anger influences discrete trust behavior, as well as the role that gender and social distance play in this relationship. Hoffman et al. (1998) suggest that the issue of national and cultural differences in trust is one we need to understand better; thus, we further explored the cross-cultural generalization of the proposed functional relationship.

We found evidence to support the conditions by which anger influences trust. In two studies we demonstrated that the influence of anger on trust was moderated by gender and the social distance of the trustee. Results indicated that in both Germany and China, men’s trust behavior was influenced neither by anger nor by the social distance of trustee. Women’s trust was context-dependent and influenced by both their emotional experience and the perceived social distance of the trustee. These functional relationships were detected in both the samples from individualistic and collectivistic cultures, and thus provided support for cross-cultural generalizability. In general, women tend to be more sensitive to social cues in determining proper social behavior than are men (Anderson and Blanchard, 1982; Eagly and Wood, 1991). Therefore, social heuristics such as the information of their current emotions and the perceived social distance to others, are influential in the formation of trust action plans in women. These findings introduce new insights into the research about the gender differences in trust, as a number of studies to date found women to be more financially risk averse and thus less trusting than men (e.g., Chaudhuri and Gangadharan, 2007; Buchan et al., 2008; Charness and Gneezy, 2012), while other studies found that women trust others not less than men (e.g., Croson and Buchan, 1999; Cox and Deck, 2006; Schwieren and Sutter, 2008). It might be that the existence of gender differences in applying the social heuristics in determining trust behavior causes the inconsistent gender differences found in the trust literature. The findings here indicate that women and men may take different information processing approaches to deal with emotions in the rich emotion social contexts, which sheds light on the connection between emotion, gender and trust.

The present results also shed light on the question of when anger influences trust. Based on the AIM, anger could influence trust when people engage in heuristic processing, while the influence would not take place when people engage in analytic processing (Forgas, 1995; Dunn and Schweitzer, 2005).^3^ Therefore, we expect that the trusting person would follow the heuristic processing and that anger would influence trust when the trustee is an absolute stranger, as the emotion (i.e., anger) is the only influential information in the trust decision-making process. In contrast, the trusting person would rely on analytic process when he/she has received some information about the trustee; in our experiment, allowing the two individuals to engage in some interaction in the context of the online chatting eliminated the effect of anger on trust that was observed in the no-chat condition. Our results from Germany, but not from China, provide support for these assumptions. We propose two possible explanations for these inconsistent results based on the evidence from cross-cultural studies.

The first explanation accounts for the differences of cultural norm of anger experiences. Anger is normative in the European-American cultures, where it presumptively promotes independence, and undesirable in East Asian cultures, where it presumably violates the goal of relational harmony (Kitayama et al., 2006; Boiger et al., 2013). Therefore, German women were driven by their anger and followed the affect heuristic to make a faster decision and to invest more money to a complete stranger. Analytic processing, on the other hand, was followed when the trustee was someone who they have communicated with, and this consequently made their decision-making process slower and eliminated the positive effect of anger on trust. However, anger suppression is encouraged in the collectivistic culture in order to maintain the interpersonal harmony (Mesquita and Walker, 2003; Kitayama et al., 2006; Butler et al., 2007; Maxwell et al., 2007; Boiger et al., 2013), especially in Chinese women as compared to Chinese men (Tanzer et al., 1996).^4^ They are able to maintain intact social connections by suppressing their anger, such that any negative impacts on the social interaction and the relationship is avoided (Butler et al., 2007; Boiger et al., 2016). In this case, regardless of whether the trustee is an absolute stranger or someone whom they have chatted with, Chinese women might suppress their anger and rely on analytic processing, a relatively slow process, as indicated by their response times. Consequently, they would invest similar amounts of money in their counterparts in the anger and in the control situations.

Second, the different cultural orientations between individualist and collectivist cultures may provide an explanation for the inconsistent influence of the social distance on trust. In individualist cultures, the goals of individual have priority over those of the group; while in a typical collectivist country, such as China, the needs of group take precedence over the needs of individual (Hofstede, 2001; Oyserman et al., 2002). These differences may also explain the cultural variation in how groups are formed and maintained. In Western Europe, groups tend to be temporary and flexible, and could be formed by the creation of artificial groups for a given purpose and exited when the purpose has been fulfilled (Triandis, 1995). In collectivist cultures, on the other hand, ingroups are more permanent, one is typically born into these groups, and group departure is often not optional, as is the case with natural groups like family or the same university. These cultural differences regarding how groups are formed and maintained might cause differences in the way individuals trust others (Triandis, 1994; Buchan et al., 2006). Buchan et al. (2006) found a group-based bias in trust behavior, such that Americans invested more in ingroup members than to outgroup members. In China, in contrast, more was invested to outgroup partners than to ingroup partners. Following the same method as Buchan et al. (2006) in manipulating social distance, results from our studies indicate that this group-based bias in trust might be true for women but not for men. German women in the control condition tended to send more money to someone from their chatting group (ingroup member) than to an absolute stranger (outgroup member), because the decreasing social distance participants perceived produced a bias in their trust behavior. However, this experimentally promoted ingroup bias did not exist in China when the social distance was manipulated through the creation of artificial groups but not based on natural groups. Being different from German women, Chinese women perceived no significant decrease of social distance to their game partner after the manipulation of social distance, as they might have realized that an absolute stranger in the no-chat condition might have been from the same university as them, thus being a part of their natural group. Therefore, the greater sums of money sent to an absolute stranger than to someone from the chatting group by the Chinese women might indicate resistance to the artificial group boundary, and point to the tendency to remain close to their perceived natural group (Chen and Kenrick, 2002; Buchan et al., 2006). These potential cross-cultural differences of group creation and perceived social distance may have imperceptible influences on people’s emotion and trust behavior, which is not yet clear and deserves further investigation.

## Limitations and Future Directions

This research has several limitations. First, our samples consisted of students, which may be more similar across cultures than, for example, members of the general population (Boiger et al., 2014). Therefore, our findings may have underestimated cultural differences in the effects of anger, the social distance of the trustee, and gender on trust. Second, we relied on samples from a limited cross-cultural scope. This study was conducted with participants from two countries, namely Germany and China, which represent a Western European culture and an Eastern Asian culture, respectively. These cultures are known to differ on a variety of dimensions, among which the differentiation between individualism and collectivism is probably most prominent (Triandis, 1994, 1995; Hofstede, 2001). Thus, comparing samples differing on this dimension in order to explore cultural influences might be reasonable. Still, one has to keep in mind that our conclusions are drawn based on samples from only two countries. Therefore, further replications should be conducted with samples from other countries. Third, the manipulation of social distance (chatting task) itself may have created some bias on people’s trust in general (not only to their interaction partner whom they later matched with) or may introduce a bias of bonding with others. The future study may focus on the function of social distance by providing interaction task for all participants and then match them in the trust game with people either from the interaction group or complete strangers.

The final limitation involves the influence of the emotional experience on the trust process. This study focuses on anger but not on other emotions, providing evidence as to the conditions by which anger influences trust via the role of gender and social distance. Future research could investigate whether these conditions can be generalized to other emotional contexts. For instance, future research should investigate whether discrete emotions such as anger (high certainty, and associated approach motivation) and fear (low certainty, and associated avoidance motivation) differentially affect trust behavior.

## Conclusion

In the two studies outlined in this article we demonstrated that the influence of anger on trust was moderated by gender and the perceived social distance of the trustee. Results revealed that in both Germany and China, men’s trust was influenced neither by anger nor by social distance. The pattern for women differed by country. Consistent with the theories of ATF (Lerner and Keltner, 2000, 2001) and AIM (Forgas, 1995), the trust of German women was increased by anger based on the affect heuristic processing, whereas analytic processing might have been followed when the trustee was someone who women have communicated with, which consequently eliminated the positive effect of anger on trust. In the Chinese culture, on the other hand, anger suppression is a highly important personality trait that maintains interpersonal harmony (Kitayama et al., 2006; Butler et al., 2007; Maxwell et al., 2007; Boiger et al., 2013). Thus, Chinese women in our study appeared to suppress their anger and utilize analytic processing, investing a similar amount of money to the trustee in the anger condition as in the control situation, regardless of whether the trustee was an absolute stranger or someone that they had chatted with. In conclusion, women’s trust was indicated to be more context-dependent, differing based on the experience of their anger and the social distance of the trustee than men’s. These functional relationships were detected in both the samples from individualistic and collectivistic cultures, and thus provide basis for cross-cultural generalizability.

## Data Availability Statement

The original contributions generated for this study are included in the article/Supplementary Material, further inquiries can be directed to the corresponding author.

## Ethics Statement

The studies involving human participants were reviewed and approved by University of Konstanz and Qingdao University. The patients/participants provided their written informed consent to participate in this study.

## Author Contributions

KZ, TG, and FC conceived and designed the experiments. KZ performed the experiments and literature research. KZ and TG analyzed the data. KZ and FC contributed materials and analysis tools. KZ, TG, and AS wrote the manuscript. All authors contributed to the article and approved the submitted version.

## Conflict of Interest

The authors declare that the research was conducted in the absence of any commercial or financial relationships that could be construed as a potential conflict of interest.

## Footnotes

1 The rating was reverse coded from 1 (large social distance) to 7 (small social distance) for a better understanding of social distance.

2 The average payment in this experiment was equivalent about 4.5 Euro in China, which was 3.2 Euro less than in Germany, as the average payment (about 2.0 Euro/hour) in China was relative lower than the one in Germany (about 8.0 Euro/hour).

3 We compared the response time in Study 1 and Study 2. German women made faster decision than Chinese women in the anger_no chat condition (Kolmogorov–Smirnov test, *Z* = −2.38, *p* = 0.017, two-sided, Bootstrap samples = 10,000), but not in the other three conditions (*p*s > 0.05), which might indicate that German women followed the affect heuristic and made rapid decisions. As compared with German and Chinese men, there were no significant differences in response time across the four experimental conditions, and were quite similar pattern of response time in both samples (*p*s > 0.05).

4 We calculated the response time of men and women between the anger and control conditions in Study 2, and found Chinese men tended to make faster decision in anger conditions (anger_no chat, anger_chat) than in control conditions (control_no chat, control_chat) (Kolmogorov–Smirnov test, *Z* = 1.18, *p* = 0.048, one-sided, Bootstrap samples = 10,000), while no differences were observed between Chinese women in these two conditions (Kolmogorov–Smirnov test, *Z* = 0.50, *p* = 0.47, one-sided, Bootstrap samples = 10,000).
