# Supplementary material for: The Differential Effects of Anger on Trust: A Cross-Cultural Comparison of the Effects of Gender and Social Distance
Source: Front Psychol. 2020 Dec 23;11:597436. doi: 10.3389/fpsyg.2020.597436 (PMC7786058; doi:10.3389/fpsyg.2020.597436)
Supplement: Supplementary file 3 [file Table_3.DOCX]

***Appendix 1: Pilot study 1***

We recruited 32 German university students (78.1% female, age: *M*=23.4 years, *SD* = 2.75) in a pilot study to test the efficacy of the adopted AEMT in inducing anger. Participants completed the emotion rating survey before and after writing the AEMT (pre- and post-emotion ratings, respectively). Anger (the target emotion) and other nine emotions (anxiety, fear, sadness, shame, hopelessness, boredom, enjoyment, pride, and hope) were assessed, in order to be able to judge whether our manipulation would indeed have an effect on experienced anger. In the baseline, there were no significant group differences in anger between the anger and control conditions, *t*(30) = -1.67, *p* = .105 (Anger: *M* = 0.15, *SD* = 0.27; Control: *M* = 0.46, *SD* = 0.70), and there were also no significant group differences in the other nine emotions (*p* > .05 in all the *t*-tests). Following the anger manipulation, participants in the anger condition showed significantly higher levels of anger than the participants in the control condition, *t*(30) = 3.06, *p* < .01 (Anger: *M* = 1.90, *SD* = 1.54; Control: *M* = 0.58, *SD* = 0.77), while there were nonsignificant group differences with respect to the other nine emotions (*p* > .05 in all the *t*-tests).

***Appendix 2: Pilot study 2***

Before replicating the main experimental study, a pretest similar to the German pilot study was applied to test the efficacy of the adopted AEMT in inducing anger in Chinese participants. We recruited 32 Chinese university students (75% female, age: *M*=20.5 years, *SD* = 1.85). Participants completed the emotion rating survey before and after writing the AEMT (pre- and post-emotion ratings, respectively). Anger (the target emotion) and other nine emotions (anxiety, fear, sadness, shame, hopelessness, boredom, enjoyment, pride, and hope) were assessed. In the baseline, there were no significant group differences in anger between the anger and control conditions, *t*(30) = -1.02, *p* = .315 (Anger: *M* = 0.21, *SD* = 0.42; Control: *M* = 0.46, *SD* = 0.89), and there were also no significant group differences with respect to the other nine emotions (*p*s > .05 in all the *t*-tests). Following the anger manipulation, participants in the anger condition showed significantly higher levels of anger than the participants in the control condition, *t*(30) = 4.01, *p* < .001 (Anger: *M* = 0.75, *SD* = 0.67; Control: *M* = 0.06, *SD* = 0.13), There were also significantly higher levels of shame in the anger condition than in the control condition *t*(30) = 2.48, *p* < .05 (Anger: *M* = 0.66, *SD* = 0.57; Control: *M* = 0.23, *SD* = 0.42). No significant differences were observed between the levels of shame from t1 to t2 in both conditions, *F*(1, 30) = 0.79, *p* > .05. Additionally, there were nonsignificant group differences in the rest eight emotions (*p*s > .05 in all the *t*-tests). Therefore, we concluded that only anger was aroused following by the AEMT. These results were comparable to those of the German sample and provided evidence for the applicability of the experimental design to Chinese participants which we considered as a prerequisite for conducting Study 2.
